# Frequency and prognostic impact of basic critical care echocardiography abnormalities in patients with acute respiratory distress syndrome

**DOI:** 10.1186/s13613-017-0343-9

**Published:** 2017-12-19

**Authors:** Kay Choong See, Jeffrey Ng, Wen Ting Siow, Venetia Ong, Jason Phua

**Affiliations:** 1 Division of Respiratory and Critical Care Medicine, University Medicine Cluster, National University Health System, 1E Kent Ridge Road, NUHS Tower Block Level 10, Singapore, 119228 Singapore; 2 Department of Medicine, Yong Loo Lin School of Medicine, National University of Singapore, Singapore, Singapore

**Keywords:** Echocardiography, Intensive care units, Respiratory distress syndrome, adult, Cor pulmonale, Ventricular dysfunction, left

## Abstract

**Background:**

Among intensive care unit (ICU) patients with acute respiratory distress syndrome (ARDS), apart from acute cor pulmonale (ACP), the frequency and prognostic impact of basic critical care echocardiography (BCCE) abnormalities are not well defined.

**Methods:**

Observational study of patients with ARDS, admitted from September 2012 to May 2014, who underwent BCCE within 48 h of admission to a 20-bed medical ICU. We examined the association of two major BCCE-detected abnormalities (left ventricular ejection fraction < 40% and severe ACP) with ICU/hospital mortality and ICU/hospital length of stay. Multivariable models adjusted for age and illness severity.

**Results:**

Of 234 patients with ARDS (age 62.3 ± 14.3 years; 88/37.6% female; APACHE II 26.8 ± 8.3; 26.5% ICU mortality; 32.1% hospital mortality), 94 (40.2%) had at least one major BCCE-detected abnormality. The more common major BCCE abnormality found was severe ACP (28.2%), followed by left ventricular ejection fraction < 40% (16.2%). On multivariate analysis, only severe ACP remained significantly associated with ICU/hospital mortality. Hospital mortality for mild, moderate and severe ARDS was 17.0, 27.9 and 50.0%, respectively (without severe ACP), and was 29.2, 48.3 and 53.8%, respectively (with severe ACP).

**Conclusions:**

BCCE abnormalities were common, but only severe ACP had prognostic significance in ARDS, identifying patients who are at increased risk of ICU and hospital mortality. The presence of severe ACP appears to upstage ARDS severity by one level.

## Background

Acute respiratory distress syndrome (ARDS) is a common critical illness with high mortality [1]. Patients may develop cardiac complications as the result of severe illness or as a side effect of treatment. In particular, patients with ARDS may develop right ventricular overload and acute cor pulmonale (ACP) [2–4]. The pathophysiology of ACP is complex and is related to pulmonary vasoconstriction, permissive hypercapnia, intrapulmonary microthrombi and positive pressure mechanical ventilation [4–6].

The spread of basic critical care echocardiography (BCCE) technology and expertise will allow intensive care unit (ICU) physicians to incorporate BCCE into routine clinical practice. For instance, BCCE has been recognized as a useful tool for hemodynamic optimization of patients known to have shock [7, 8]. BCCE can also be used to detect two major echocardiographic abnormalities: left ventricular ejection fraction and severe ACP [9].

Apart from ACP, there is uncertainty over the frequency and prognostic significance of the above-mentioned two major BCCE abnormalities in newly admitted ICU patients with ARDS. Knowing this information would be useful for delineating the potential role of BCCE screening for such critically ill patients, which is currently unclear [10]. We therefore aimed to investigate the frequency of BCCE-detected abnormalities in patients with ARDS and to elucidate any associations with ICU and hospital mortality, or with increased ICU or hospital length of stay.

## Methods

### Participants and setting

We conducted a prospective observational study of patients with ARDS admitted to our 20-bed medical ICU from September 2012 to May 2014, who underwent BCCE within 48 h of admission. Due to manpower constraints, BCCE was routinely scheduled on three weekdays (Monday, Wednesday and Friday) and was done on an ad hoc basis over weekends and public holidays for newly admitted patients. BCCE was not done if patients discharged or died before it could be done. Three patients, who had surgical dressings over the chest/abdomen precluding satisfactory transthoracic echocardiographic windows, were excluded. No other exclusion criteria were used. Only the first ICU admission for each patient was analysed. The presence and severity of ARDS were determined on the day of BCCE.

Our Ethics Review Board (National Healthcare Group Domain-Specific Review Board) permitted waiver of informed consent (DSRB B/2013/00132). Although we reported some of our data in a prior study on BCCE training [9], our current study has different aims, the number of ARDS patients has been doubled and we have collected new information pertaining to ARDS.

### Scanning procedure and clinical care

**Fig. 1:**
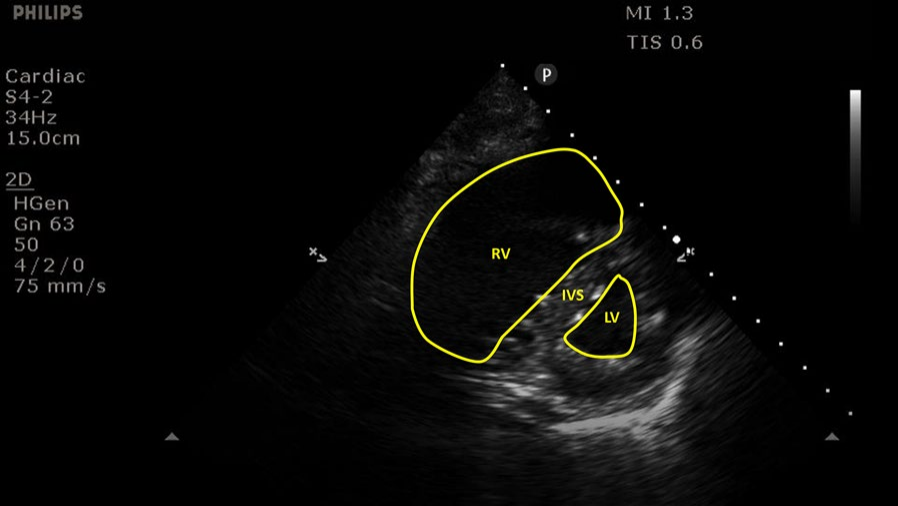
An example of acute cor pulmonale. 82-year-old woman with myelodysplastic syndrome was intubated for severe community-acquired pneumonia. She had no known cardiac problems or chronic lung disease. On admission to the intensive care unit, basic echocardiography was done. The parasternal short axis view showed a dilated right ventricle (RV) in end diastole at the papillary muscle level and interventricular septum (IVS) straightening, indicating acute cor pulmonale. The RV and left ventricle (LV) areas may be determined via endocardial tracing as shown, though in many cases, an RV/LV ratio of ≥ 1 can be determined visually without routine manual tracing

BCCE was only performed by competent attending physicians or fellows, or by fellows under supervision, using the Sparq Ultrasound System (Philips Healthcare, Andover, MA) equipped with a 2–4 MHz broadband sector, phased array transducer. Physicians were deemed to be competent if they had at least 1 year of daily experience with BCCE performance and interpretation. At least seven standard views (acoustic windows) were obtained and recorded for each BCCE scan: parasternal long axis, parasternal short axis, apical four-chamber (three views), subcostal and inferior vena cava (IVC) [9]. For this study, we considered two major abnormalities because these are reliably detected by bedside echocardiography: visually estimated left ventricular ejection fraction < 40% and severe ACP (right ventricular dilatation with the right-to-left ventricular size (area) ratio ≥ 1 in end diastole at the papillary muscle level and interventricular septal straightening/paradoxical motion using the parasternal short axis view) [9, 11, 12]. The presence of ACP was determined by visually examining the relative sizes of the right and left ventricles in both the parasternal short axis views and the apical four-chamber views. We chose the parasternal short axis view as the main view to assess the relative sizes of the right and left ventricles and to assess for septal straightening/paradoxical motion (see Fig. 1 for an example), as this view had a fixed landmark (papillary muscles) and was not prone to foreshortening or rotational error. Since we relied on a ratio of 1 between the right and left ventricle sizes, we also found that visual comparison was rapid and accurate without routine manual tracing of the endocardial borders. We used the apical four-chamber as a secondary safeguard against false ACP determination, which in our experience did not occur. Conversely, if we had used the apical four-chamber view as the main view, potential foreshortening or rotational error would lead to under-recognition of RV dilatation and ACP. We determined that the cor pulmonale was acute, rather than chronic, if there was no significant right ventricular dysfunction noted previously, either clinically or from prior echocardiography.

Usage of BCCE findings and clinical care were left to the discretion of the attending physicians. During the time period of the study, we did not enforce any protocol with regard to specific BCCE abnormalities or mandate repeat scanning. For hemodynamic management, our ICU relied on arterial line blood pressure [13], with the option of FloTrac/Vigileo (Edwards Lifesciences, Irvine, CA) continuous arterial pressure waveform-based cardiac output measurements and echocardiography. Noradrenaline was the preferred vasopressor, particularly for septic shock [14]. For ventilator management, patients received low tidal volume ventilation with minimal analgesia and sedation, but not high-frequency oscillatory ventilation [15] or nitric oxide [16]. None of the patients received prone positioning as this was not usual practice for our ICU during the study period. Sepsis was treated with early, broad-spectrum antibiotics and source control.

### Data collection

Clinical data were extracted from the ICU computerized database and medical records. The latest arterial blood gas measurement, which was taken on the day of the BCCE scan, was used to compute the arterial oxygen partial pressure (in mmHg) to inspired oxygen fraction (PF ratio). Respiratory parameters extracted from the database included tidal volume, respiratory rate and positive end-expiratory pressure at the time of BCCE. We did not routinely perform inspiratory pause manoeuvres at the time of BCCE. These manoeuvres were nonetheless done routinely for mechanically ventilated patients every morning by our respiratory therapists, and we recorded plateau pressures and respiratory compliance readings taken on the morning of the BCCE scan.

ARDS was defined using the Berlin Definition [17]. To diagnose ARDS, patients needed a PF ratio < 300 mmHg, a positive end-expiratory pressure ≥ 5 cm H_2_O (may be non-invasively delivered for mild ARDS), no predominant cardiac failure or fluid overload and bilateral infiltrates on chest radiography. These features must also develop within 1 week of a known clinical insult or new or worsening respiratory symptoms. The severity of ARDS was classified as mild (PF ratio 201–300 mmHg), moderate (PF ratio 101–200 mmHg) and severe (PF ratio 100 mmHg or less).

### Statistical analysis

Univariate comparisons of proportions, means and medians were, respectively, done using Fisher exact, Student t and Wilcoxon rank-sum tests. Confidence intervals of binomial probability distributions were computed using the Clopper–Pearson (exact) method. We examined the association of major BCCE-detected abnormalities with ICU/hospital mortality and ICU/hospital length of stay. Logistic regression was used to analyse mortality as an outcome. Linear regression was used to analyse length of stay (log-transformed to achieve normality) as an outcome. Both the logistic regression and linear regression models corrected for age, and Acute Physiology and Chronic Health Evaluation (APACHE) II score. Statistical significance was taken as *P* < 0.05.

## Results

During the study period, 1218 patients (1371 admissions; age 61.8 ± 16.5 years; 492/40.4% female; APACHE II 22.7 ± 9.0) were treated in our ICU. We performed BCCE for 651 patients (709 admissions; age 61.0 ± 16.6 years; 251/38.6% female; APACHE II 24.0 ± 8.2). Although we did not collect information on the presence or absence of ARDS for patients who did not receive BCCE, these patients did not differ from the whole ICU population in terms of age (*P* = 0.973), gender (*P* = 0.448) and APACHE II score (*P* = 0.915).

Among patients who received BCCE, 234 patients fulfilled the Berlin Definition of ARDS (age 62.3 ± 14.3 years; 88/37.6% female; APACHE II 26.8 ± 8.3) (Table 1). Ninety-four patients (40.2%) had at least one major BCCE-detected abnormality. The more common major BCCE abnormality found was severe ACP (28.2%), followed by left ventricular ejection fraction < 40% (16.2%). Among our 66 patients with ACP, no patient had any clinical cor pulmonale at baseline. Additionally, of 42 (63.6% of 66) patients who had prior transthoracic echocardiography, no patient had moderate or severe right ventricular dysfunction noted, and only 3 (4.6% of 66) patients had mild right ventricular dysfunction noted. Hospital mortality was 20.5% (83 patients, 95% CI 12.4–30.8%) for mild ARDS, 33.3% (108 patients, 95% CI 24.6–43.1%) for moderate ARDS and 51.2% (43 patients, 95% CI 35.5–66.7%) for severe ARDS (Table 2).

**Table 1:** Patient characteristics and outcomes

Patient characteristic or outcome	All patients with ARDS (*N* = 234)	Patients with ARDS, without severe ACP (*N* = 168)	Patients with ARDS, with severe ACP (*N* = 66)	*P* value
Age (years) (mean ± SD)	62.3 ± 14.3	62.0 ± 14.7	64.8 ± 12.9	0.180
Female sex (%)	88 (37.6)	65 (38.7)	23 (34.9)	0.654
APACHE II score (mean ± SD)	26.8 ± 8.3	26.7 ± 8.1	27.3 ± 8.7	0.615
Arterial blood gas measurement				
PF ratio (mmHg) (mean ± SD)	171 ± 67	172 ± 69	169 ± 63	0.801
pH (mean ± SD)	7.33 ± 0.12	7.34 ± 0.11	7.31 ± 0.13	0.065
PaCO_2_ (mmHg) (mean ± SD)	43 ± 14	42 ± 13	47 ± 16	0.001
ARDS^a^ (%)				
Mild	83 (35.5)	59 (35.1)	24 (36.4)	0.892
Moderate	108 (46.2)	79 (47.0)	29 (43.9)	
Severe	43 (18.4)	30 (17.9)	13 (19.7)	
Primary diagnosis (%)				
Pneumonia	208 (88.9)	151 (89.9)	57 (86.4)	0.489
Non-pneumonia sepsis	26 (11.1)	17 (10.1)	9 (13.6)	
Comorbidities (%)				
Diabetes mellitus	81 (34.6)	61 (36.3)	20 (30.3)	0.446
Hypertension	115 (49.2)	86 (51.2)	29 (43.9)	0.384
Ischaemic heart disease	55 (23.5)	43 (25.6)	12 (18.2)	0.304
Chronic heart failure	9 (3.9)	7 (4.2)	2 (3.0)	1.000
Asthma	14 (6.0)	10 (6.0)	4 (6.1)	1.000
COPD	17 (7.3)	13 (7.7)	4 (6.1)	0.785
Bronchiectasis	10 (4.3)	7 (4.2)	3 (4.6)	1.000
Chronic renal failure	38 (16.2)	27 (16.1)	11 (16.7)	1.000
Chronic liver disease	10 (4.3)	8 (4.8)	2 (3.0)	0.729
Stroke	16 (6.8)	13 (7.7)	3 (4.6)	0.566
Cancer	39 (16.7)	31 (18.5)	8 (12.1)	0.330
Actual body weight (kg) (mean ± SD)	63.3 ± 17.2	63.4 ± 16.6	62.9 ± 18.6	0.840
Ventilation modes (%)				
Nil ventilation	0 (0.0)	0 (0.0)	0 (0.0)	0.942
CPAP	17 (7.3)	13 (7.7)	4 (6.1)	
NIV	10 (4.3)	7 (4.2)	3 (4.6)	
Invasive	207 (88.5)	148 (88.1)	59 (89.4)	
Respiratory parameters at time of BCCE				
Respiratory rate (breaths/min) (mean ± SD)	24 ± 3	24 ± 4	24 ± 2	0.274
Tidal volume (ml) (mean ± SD)	408 ± 113	409 ± 113	407 ± 112	0.931
Tidal volume (ml/kg IBW) (mean ± SD)	7 ± 2	7 ± 2	7 ± 3	0.276
PEEP (cm H2O) (mean ± SD)	7 ± 3	6 ± 3	7 ± 3	0.089
Plateau pressure^b^ (cm H_2_O) (mean ± SD)	21 ± 3	21 ± 2	21 ± 5	0.507
Compliance^b^ (ml/cm H_2_O) (mean ± SD)	31 ± 14	29 ± 12	34 ± 18	0.047
On vasoactive agents (%)				
Any agent^c^	77 (32.9)	55 (32.7)	22 (33.3)	1.000
Dopamine	4 (1.7)	2 (1.2)	2 (3.0)	0.316
Noradrenaline	73 (31.2)	52 (31.0)	21 (31.8)	1.000
Dobutamine	2 (0.9)	2 (1.2)	0 (0.0)	1.000
Vasopressin	1 (0.4)	1 (0.6)	0 (0.0)	1.000
BCCE-detected major abnormalities (%)				
Left ventricular ejection fraction < 40%	38 (16.2)	28 (16.7)	10 (15.2)	0.846
Severe acute cor pulmonale	66 (28.2)	0 (0.0)	66 (100.0)	< 0.001
Any major abnormalities	94 (40.2)	28 (16.7)	66 (100.0)	< 0.001
LOS, ICU (days), median (IQR)	7 (4–12)	7 (4–12)	7 (3–13)	0.931
LOS, hospital (days), median (IQR)	17.5 (9–28)	18 (9–26)	17 (8–31)	0.837
Mortality, ICU (%)	62 (26.5)	37 (22.0)	25 (37.9)	0.021
Mortality, hospital (%)	75 (32.1)	47 (28.0)	28 (42.4)	0.043

*ACP* Acute cor pulmonale (severe ACP is defined as right ventricular dilatation with the right-to-left ventricular size ratio ≥ 1 in end diastole at the papillary muscle level and interventricular septal straightening/paradoxical motion), *APACHE II* Acute Physiology and Chronic Health Evaluation II, *ARDS* acute respiratory distress syndrome, *BCCE* basic critical care echocardiography, *COPD* chronic obstructive pulmonary disease, *CPAP* continuous positive airway pressure, *IBW* ideal body weight. For males, IBW = 50 + 2.3 kg for each increment of 2.54 cm (1 inch) in length over 152.4 cm (5 feet). For females, IBW = 45.5 + 2.3 kg for each increment of 2.54 cm (1 inch) in length over 152.4 cm (5 feet), *ICU* intensive care unit, *IQR* interquartile range, *LOS* length of stay, *PEEP* positive end-expiratory pressure, *SD* standard deviation, *NIV* non-invasive ventilation

^a^ARDS severity according to the Berlin Definition: mild (PF ratio 201–300 mmHg), moderate (PF ratio 101–200 mmHg) and severe (PF ratio 100 mmHg or less), where PF ratio is the ratio of arterial oxygen partial pressure (mmHg) to inspired oxygen fraction

^b^Data only for the intubated patients (*N* = 207)

^c^Patients could be on more than one vasoactive agent

**Table 2:** Hospital mortality of patients with acute respiratory distress syndrome, with or without severe acute cor pulmonale

Hospital mortality	All ARDS patients (*N* = 234)	ARDS patients without severe ACP (*N* = 168)	ARDS patients with severe ACP (*N* = 66)	*P* value^b^
Severity of ARDS^a^ (%, CI)				
Mild	17/83 (20.5, 12.4–30.8)	10/59 (17.0, 8.4–29.0)	7/24 (29.2, 12.6–51.1)	0.239
Moderate	36/108 (33.3, 24.6–43.1)	22/79 (27.9, 18.3–39.1)	14/29 (48.3, 29.4–67.5)	0.065
Severe	22/43 (51.2, 35.5–66.7)	15/30 (50.0, 31.3–68.7)	7/13 (53.8, 25.1–80.8)	1.000
Overall cohort (%, CI)	75/234 (32.1, 26.1–38.4)	47/168 (28.0, 21.3–35.4)	28/66 (42.4, 30.3–55.2)	0.043*

*ACP* Acute cor pulmonale (severe ACP is defined as right ventricular dilatation with the right-to-left ventricular size ratio ≥ 1 in end diastole at the papillary muscle level and interventricular septal straightening/paradoxical motion), *ARDS* acute respiratory distress syndrome, *CI* 95% confidence interval

*P < 0.05

^a^ARDS severity according to the Berlin Definition: mild (PF ratio 201–300 mmHg), moderate (PF ratio 101–200 mmHg) and severe (PF ratio 100 mmHg or less), where PF ratio is the ratio of arterial oxygen partial pressure (mmHg) to inspired oxygen fraction

^b^Computed for the mortality difference between patients with and without severe acute cor pulmonale, using the Fisher exact test

On multivariate analysis, among the major BCCE abnormalities, only severe ACP was associated with ICU and hospital mortality (Table 3). No associations between major BCCE abnormalities and ICU/hospital length of stay existed (Table 4). Hospital mortality for mild, moderate and severe ARDS was 17.0, 27.9 and 50.0%, respectively (without severe ACP), and was 29.2, 48.3 and 53.8%, respectively (with severe ACP) (Table 2).

**Table 3:** Association of basic critical care echocardiography screening-derived abnormalities with mortality in patients with acute respiratory distress syndrome

Major BCCE-detected abnormalities	ICU mortality		Hospital mortality	
	Univariate OR (95% CI)^a^	Multivariate OR (95% CI)^b^	Univariate OR (95% CI)^a^	Multivariate OR (95% CI)^b^
Screened patients with acute respiratory distress syndrome (Berlin Definition)				
Left ventricular ejection fraction < 40%	2.37 (1.15–4.89)*	2.10 (0.99–4.43)	2.19 (1.08–4.45)*	2.00 (0.95–4.18)
Severe acute cor pulmonale	2.16 (1.17–4.00)*	2.14 (1.13–4.04)*	1.90 (1.05–3.43)*	1.89 (1.02–3.50)*

*BCCE* Basic critical care echocardiography, *CI* confidence interval, *ICU* intensive care unit, *OR* odds ratio

*P < 0.05

^a^Odds ratio (with 95% confidence interval) derived using logistic regression on mortality, unadjusted

^b^Odds ratio (with 95% confidence interval) derived using multiple logistic regression on mortality, adjusted for age and Acute Physiology and Chronic Health Evaluation II score

**Table 4:** Association of basic critical care echocardiography screening-derived abnormalities with log(length of stay) in patients with acute respiratory distress syndrome

Major BCCE-detected abnormalities	Log (length of stay, ICU)		Log (length of stay, hospital)	
	Univariate ratio (95% CI)^a^	Multivariate ratio (95% CI)^b^	Univariate ratio (95% CI)^a^	Multivariate ratio (95% CI)^b^
Screened patients with acute respiratory distress syndrome (Berlin Definition)				
Left ventricular ejection fraction < 40%	1.00 (0.75–1.33)	1.00 (0.74–1.34)	0.84 (0.62–1.15)	0.86 (0.63–1.17)
Severe acute cor pulmonale	1.07 (0.84–1.36)	1.10 (0.87–1.39)	1.04 (0.81–1.34)	1.06 (0.82–1.37)

*BCCE* Basic critical care echocardiography, *CI* Confidence interval, *ICU* Intensive care unit

^a^Exponentiated coefficient (with 95% confidence interval) derived using linear regression on the log-transformed LOS, unadjusted

^b^Exponentiated coefficient (with 95% confidence interval) derived using multiple linear regression on the log-transformed LOS, adjusted for age and Acute Physiology and Chronic Health Evaluation II score

## Discussion

The main findings of our study are as follows: firstly, BCCE abnormalities in ARDS patients were common, affecting 40% of the patients. Secondly, the presence of severe ACP—but not moderate/severe left ventricular dysfunction—within 48 h of ICU admission identified patients who were at increased risk of ICU and hospital mortality. Thirdly, both the BCCE-detected major abnormalities were not associated with ICU or hospital length of stay. Finally, the presence of severe ACP appears to upstage ARDS severity by one level.

Our study provided new information on the relative frequency of two major BCCE abnormalities in ARDS patients. We found that the more common abnormality was severe ACP, at 28.2%, which was around the same frequency demonstrated in another smaller study [18] and in a separate study focusing on severe H1N1 infection [19]. Nonetheless, this frequency was higher than the prevalence of severe ACP of 7% found in a prior multicentre study by Mekontso-Dessap and colleagues [3], which could be due to our non-adoption of strategies such as prone positioning to off-load the right ventricle and a higher proportion (89 vs. 40%) of pneumonia (pneumonia being a risk factor for ACP) in our cohort [5]. Across the ARDS severity gradient, ACP was fairly consistently found in 28.9, 26.9 and 30.2% of mild, moderate and severe ARDS cases. This would imply that, in our setting of high pneumonia prevalence, there was little interaction between ACP and ARDS severity. It is possible that ACP may be more a consequence of treatment strategies than disease manifestation, which would then make ACP potentially modifiable. Interestingly, among patients with ARDS, patients with ACP had slightly better respiratory system compliance compared to patients without ACP. This could reflect the lung recruitment effect of slightly higher positive end-expiratory pressure applied in cases of ACP.

Moderate/severe left ventricular dysfunction was less common in our study population, occurring in 16.2% of patients. Although previously published ARDS-specific data are not available, the frequency of left ventricular dysfunction in our cohort is consistent with prior data derived from patients with septic shock [20]. Similarly, our finding that left ventricular dysfunction had no association with mortality is also consistent with the lack of association in septic patients [21–23]. Given that we saw no increased mortality even though we only used inotropic medications very sparingly, our findings do not support the need to treat isolated left ventricular dysfunction in ARDS.

In contrast to left ventricular dysfunction, we found the presence of severe ACP to be particularly important for predicting mortality [3, 4, 11, 12]. Our definition of severe ACP involved a right-to-left ventricular size ratio ≥ 1 on transthoracic echocardiography, which corresponds to the definition of severe ACP on trans*esophageal* echocardiography in a recent study [3]. The latter study also found that less severe ACP (i.e. right-to-left ventricular size ratio > 0.6 and < 1) was conversely *not* associated with mortality. The increased mortality engendered by severe ACP may be due to an increased incidence of circulatory failure in ARDS patients [4]. In our experience, such patients are harmed by fluid administration and often require moderate-to-high doses of noradrenaline support [5]. Separately, the absence of any relationship between BCCE findings and ICU/hospital length of stay is in line with prior data for ACP [4] and with our earlier study [9], which implies that length of stay may be more influenced by non-cardiac factors. Moreover, although ACP could be contributed by volume overload, we feel that this would be partially mitigated by our ICU’s fluid management protocol, which we had established since 2011 [13]. Also, although we cannot completely exclude a cardiac contribution to ACP, the overlap between left ventricular ejection fraction < 40% and ACP was only 10 patients, which was 15.2% of the 66 patients with ACP. We also did a sensitivity analysis for the presence or absence of left ventricular ejection fraction < 40%, using logistic regression with respect to ICU and hospital mortality, adjusted for age and APACHE II score. Including patients with left ventricular ejection fraction < 40%, ACP was associated with ICU and hospital mortality with an adjusted odds ratio of 2.14 (95% CI 1.13–4.04) and 1.89 (1.02–3.50), respectively. Excluding patients with left ventricular ejection fraction < 40%, ACP was associated with ICU and hospital mortality with an adjusted odds ratio of 2.38 (95% CI 1.17–4.84) and 1.88 (0.95–3.71), respectively. Therefore, the presence of left ventricular ejection fraction < 40% did not substantially alter the conclusions of our study.

Knowledge of the presence of ACP may be key to improving the survival of ARDS patients [5, 24]. To this end, Mekontso-Dessap and colleagues found that four variables could be used to risk-stratify ARDS patients for the presence of ACP (as determined by transesophageal echocardiography within three days of ARDS diagnosis): pneumonia as a cause of ARDS, driving pressure ≥ 18 cm H2O, PF ratio < 150 mmHg and arterial carbon dioxide partial pressure ≥ 48 mmHg [3]. Among our patients (Table 1), arterial carbon dioxide partial pressure was indeed significantly higher in patients with severe ACP, though we did not detect significant differences in PF ratio, pneumonia diagnosis or driving pressure. Nonetheless, to use this four-variable risk stratification method, arterial blood gases must be drawn and that patients had to be well sedated or even paralyzed for accurate measurement of driving pressure. Furthermore, after risk stratification, confirmation by echocardiography would still be required. Based on our study, we suggest an alternative approach of directly screening *all* ARDS patients with BCCE, which we believe can be done quickly at the bedside.

In addition, we found that the presence of severe ACP can significantly add to the Berlin Definition for ARDS, and should not be taken as a mere marker of ARDS severity. Previously, the ARDS Definition Task Force reported that using the Berlin Definition, mild, moderate and severe ARDS were associated with hospital or 90-day mortality of 27% (95% CI 24–30%), 32% (95% CI 29–34%) and 45% (95% CI 42–48%), respectively [17]. In our cohort of patients with ARDS, the presence of severe ACP appears to upstage ARDS severity by one level—this may have implications on treatment thresholds and patient recruitment for future studies.

Our results suggest that screening of patients on admission, rather than waiting for clinical deterioration, would be preferable for early identification and treatment of abnormalities. For instance, the detection of severe ACP in ARDS patients should prompt strategies to protect the right ventricle. Such strategies include targeting plateau pressures below 27 cm H_2_O, maintaining adequate oxygenation and avoiding hypercarbia beyond 60 mmHg [2, 5]. Prone positioning to off-load the right ventricle and extracorporeal carbon dioxide removal to allow tidal volume (and hence plateau pressure) reduction could also be considered [5, 6]. However, while we encourage BCCE, it should only be done if frontline physicians are competent in its use and interpretation [25]. Moreover, it is a complementary modality and does not replace good clinical acumen and practice.

We acknowledge limitations for our study. Firstly, we performed a single-centre observational study, and our results require external validation. Secondly, due to resource limitations, we only managed to screen patients once within 48 h of admission and do not know whether a narrower screening interval (e.g. within 24 h of admission) or repeated screening after that would yield further information. Thirdly, we did not utilize transesophageal echocardiography as our ICU physicians have not acquired this level of expertise, and transesophageal echocardiography may improve the detection of severe ACP compared to using transthoracic echocardiography. Fourthly, although we checked that no patient had any pre-existing cor pulmonale clinically or on prior echocardiography (which was available for 63.6% of ACP cases), some patients might have developed subclinical cor pulmonale after their last echocardiography. Fifthly, we concede that determination of both LVEF and ACP may be imperfect. Nonetheless, in our experience, accuracy of visual LVEF grading and visual estimation of RV/LV size ratio were fairly good, even for trainees when compared with an experienced supervisor (correct grading achieved in 85% of cases for visual LVEF and in 92.5% of cases for visual estimation of RV/LV size ratio, after performing 30 echo examinations) [9]. Finally, we did not mask BCCE findings from clinicians, which meant that BCCE could have changed management. We did not study specific treatments administered, but should they improve survival, the association of BCCE-detected abnormalities with mortality would then be biased towards the null.

In conclusion, severe ACP—but not left ventricular dysfunction—may help identify ARDS patients at elevated risk of ICU and hospital mortality. BCCE, when used as a screening tool, can then alert the treating physician to the presence of ACP, allowing prompt institution of measures that may alter ARDS outcomes. While further validation is required, we believe that our study should encourage ICU physicians to incorporate BCCE into routine screening of ARDS patients admitted to ICU.

## Abbreviations

ACP: acute cor pulmonale
APACHE: acute physiology and chronic health evaluation
BCCE: basic critical care echocardiography
ICU: intensive care unit
PF ratio: ratio of arterial oxygen partial pressure to inspired oxygen fraction
